# Carbon nanomaterials as contrast agents for breast cancer diagnosis and therapy

**DOI:** 10.1186/2051-1426-3-S1-P5

**Published:** 2015-08-14

**Authors:** Sara Dolci, Valentina Domenici, Gianpaolo Vidili, Elisabetta Avitabile, Marco Orecchioni, Alberto Bianco, Lucia Delogu

**Affiliations:** 1 University of Pisa, Pisa, Italy; 2 University of Sassari, Sassari, Italy; 3 CNRS, Institut de Biologie Moléculaire et Cellulaire, Laboratoire d’Immunologie et Chimie Thérapeutiques, Strasbourg, France

## 

Nanotechnology is the promise to fight breast cancer (BC) more specifically and effectively [1]. In this context carbon nanomaterials (CNs) have attracted the scientific community and the public interest [2]. Common modalities for BC diagnosis are ultrasonography (US) and magnetic resonance imaging (MRI). US is the most useful modality in the evaluation of palpable BC masses that are mammographically occult in women younger than 30’s. Here we show CNs as highly and long lasting echogenic materials. Experiments on swine models confirmed that CNs are clearly visible under US and didn’t exert toxicity. In the current market dual-imaging agents are missed; here we also demonstrate the immune-compatibility and high echogenic properties in water and in whole blood of cysteine functionalized super paramagnetic nanoparticles (CY-SPION), well-known MRI agents [3]. Thanks to these findings, and the ability to load CNs to many moieties [4-6], we propose dual-contrast agents, CNs-CY-SPION conjugates, to improve BC diagnosis. Future perspectives is to conjugate CN-SPION to targeted drugs against BC. In summary, we lay the foundations for novel contrast agents, for therapy and multimodal diagnosis of BC, combining high imaging performances with unique potential therapeutic applications, such as specific targeting capabilities, drug delivery, immunotherapy and hyperthermia.
